# The Three-Dimensional Body Center of Mass at the Workplace under Hypogravity

**DOI:** 10.3390/bioengineering10101221

**Published:** 2023-10-19

**Authors:** Tatiana Maillard

**Affiliations:** Space Innovation, Swiss Federal Institute of Technology in Lausanne, 1015 Lausanne, Switzerland; tatiana.maillard@alumni.epfl.ch

**Keywords:** postural stability, reduced gravity, manual handling, biomechanics

## Abstract

The center of mass dynamics of the seated posture of humans in a work environment under hypogravity (0 < g < 1) have rarely been investigated, and such research is yet to be carried out. The present study determined the difference in the body system of 32 participants working under simulated 1/6 g (Moon) and 1 g (Earth) for comparison using static and dynamic task measurements. This was based on a markerless motion capture method that analyzed participants’ center of mass at the start, middle and end of the task when they began to get fatigued. According to this analysis, there is a positive relationship (*p* < 0.01) with a positive coefficient of correlation between the downward center of mass body shift along the proximodistal axis and gravity level for males and females. At the same time, the same positive relationship (*p* < 0.01) between the tilt of the body backward along the anterior–posterior axis and the level of gravity was found only in females. This offers fresh perspectives for comprehending hypogravity in a broader framework regarding its impact on musculoskeletal disorders. It can also improve workplace ergonomics, body stability, equipment design, and biomechanics.

## 1. Introduction

Creating a suitable workspace for astronauts is vital during the development of space missions. This should contribute to productivity and overall mental health [1]. In a microgravity (MG) and hypogravity (HG) environment, the work performance level expected in Earth settings may be reduced [2,3]. As learned from experiences in MG, HG can lead to adaptive musculoskeletal disorders [4,5], balance problems [6], cardiovascular issues [7], and vision impairment [8]. As a result of vestibular and sensory adaptation, all interfaces, including fixation systems, must be created with the consideration of the environment and gravity in which they would be utilized [3,9]. Thus, in lunar and Martian environments, the effects of HG must be examined to prevent health problems and physiological changes and to ensure the smooth adaptation of the body.

Upper limb motion under HG remains poorly understood, as most research focuses on lower limbs [10,11,12]. Few studies have explored sitting positions with operational weights under HG [13]. This knowledge gap makes it difficult to evaluate and propose mitigating measures. Compared to Earth, there is limited evidence of upper limb responses to the HG during short- or long-term missions.

Examining how the upper limbs react to HG can be achieved through the study of the center of mass (CoM). Moreover, the study of the CoM is crucial for workplace ergonomics [14,15,16,17,18] because it helps to optimize the design of work environments, equipment, and tasks to ensure the safety, comfort, and well-being of workers. The location of the CoM of a seated participant can be used for seat design, restraint systems [19], and other human-centered products and environments. Also, its understanding and consideration are important in body stability [20], posture and alignment [21], lifting and manual handling [18,22], and biomechanics [23]. An evaluation of the CoM requires a time-efficient measurement and high accuracy, especially in the case of body balance measurements [20].

The CoM is one of the main problems of biomechanics and locomotion. It helps during the modeling of the human body and its activity [24], i.e., the assessment of static positions and different kinds of movement. In general, the CoM can be measured using various direct and indirect methods. Direct methods mean that measurements can be taken directly using instruments, and indirect methods mean that they can be measured numerically using software modeling, camera data recording, or biomechanical calculations. In some situations, a laboratory approach with the reaction board on which a participant can lie can be used for indirect measurements [25]. But this requires time and the removal of the participants’ clothes. The direct approaches include methods with live subjects and cadavers [26]. In these cases, methods such as stereophotogrammetry systems and force platforms can be used [24]. Direct approaches are expensive, require sufficient measurement space, and are difficult to implement [27]. In this regard, there is a growing interest in new methods that provide ease of measurement, better accuracy, and non-invasiveness. In other cases, especially during movement, indirect methods may be useful. Indirect methods include an optical motion capture system with reflective markers [28], motion capture systems using wearable inertial measurement units (IMUs) [20], and markerless motion capture [29,30]. Due to the technical limitations of the listed methods, the markerless motion capture method is one of the most convenient and non-invasive methods for analyzing dynamic motion, especially for the underwater simulation of movements in HG conditions [31].

This study aimed to determine the CoM of the seated underwater participants to simulate HG using indirect methods, such as markerless motion capture, with 3D visualization elements. In this study, whole body CoM location analysis was assessed relative to the hip joint of participants performing static outstretched arm and dynamic weight-holding tasks in two different environments with 1/6 g and 1 g for comparison purposes. The hypothesis underlying this study is that, in the context of ergonomics and human movement analysis, individuals in hypogravity (HG) conditions will demonstrate a posterior tilt of the upper body during task engagement, as opposed to their posture in normal Earth gravity (1 g). In a broader context, this research was undertaken to identify the causes of musculoskeletal disorders in the workplace with the aim of improving safety, comfort, and productivity. It aimed to leverage modern motion analysis capabilities, namely markerless motion capture, to better quantify changes in human posture and movement non-invasively in various gravitational environments simulated in aquatic environments.

## 2. Materials and Methods

### 2.1. Experimental Setup

The experimental study included a total of 32 participants (18 males and 14 females). G*Power software was used to find the necessary number of participants for this experiment (see results in the Supplementary Materials Table S1). This study was conducted in a water tank (Swissub, Vaud, Switzerland) to simulate HG (1/6 g). Before the experiments, each participant signed a written informed form. The readiness of each participant for physical activity was evaluated through the completion of “The 2020 Physical Activity Questionnaire (PAR-Q+)” (see Supplementary Materials Figure S1) [32]. This survey had seven health-related questions with only two possible answers: “Yes” or “No.” Participants who answered “No” to all questions were eligible to participate. The Ethics Committee of the Swiss Federal Institute of Technology in Lausanne gave its approval to all of the study’s steps. The descriptive data of all participants’ anthropometric information (all experiments) are presented in Table 1. In terms of age, there were marginal differences between males and females (*p* = 0.742). Both height and weight were significantly higher for males (*p* < 0.001).

**Figure 1:**
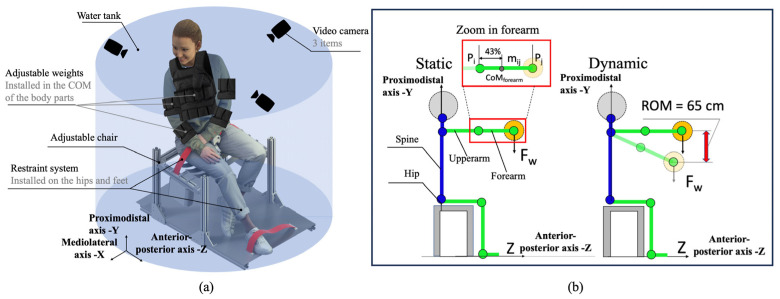
Experimental setup: (**a**) participant-adjusted chair and ballasts distributed around the body, axonometric projection; (**b**) static and dynamic tasks performed by participants with inset depicting the forearm segment, projection on the sagittal plane. ROM—range of motion. F_w_—weight of the load. The image of the female mannequin is taken from the open-source model at https://humano3D.com, accessed on 12 November 2021.

The gravity simulation was carried out with ballasts distributed on the body parts (forearm, upper arm, torso) of the participants, having the necessary buoyancy proportional to lunar gravity (g = 1.626 m/s^2^) (see Figure 1a). The heads of the participants were not submerged. To compensate for the weight of the head, an inflatable neck pillow was used, inflated to the level necessary to compensate for the weight of the participant’s head, to approximate lunar gravity. The participants were seated on a participant-adjustable chair without backrest and armrest. All participants conducted 2 types of tasks, namely static and dynamic, with two intensities: (1 kg and 3 kg) (see Figure 1b). The chosen tasks are of significant importance to astronauts because they lead to muscle fatigue and decreased performance. These include static tasks involving loads found in construction and electronics maintenance. The dynamic is related to assembly lines and component manipulation. The selected weight range (1–3 kg) corresponds to the typical instruments that astronauts regularly handle. Assessing these tasks can allow for the identification of potential injury risks and facilitate the development of countermeasures across various workplaces.

Simulating HG in aquatic environments remains challenging, especially when it comes to dynamic motion simulation. Different factors, including water resistance, the attachment of ballast to various parts of the body that affect the inertia of movement, and the ability to predict movement using the markerless motion capture method can affect the measurements. The placement of operational weights on various parts of the body has a minimal impact on the inertia of motion, primarily because the ballasts needed to simulate lunar gravity are negligible, especially on the upper arm and the forearm. The moment of inertia in this case only becomes noticeable when these ballasts exceed 3 kg, based on the moment of the inertia equation. Operating weights of this magnitude were applied only to the torso. However, since all participants performed dynamic/kinematic movements of the upper limbs with limited acceleration, and their torsos hardly changed position, the influence of the moment of inertia of the ballast due to small rotations around the reference axis cannot be considered to introduce a significant error into the final CoM calculation. The range of motion (ROM) for dynamic tasks was determined so that the influence of water resistance and wave action was minimal. It was assumed that the resistance force was less than 10% of the forearm. Thus, the maximum speed of slow hand movement was 47 cm/s [3]. The task intensity was provided with workloads held by the participants in one hand.

The modeling of lunar gravity in water conditions is based on the equation for compensation of buoyancy of body segments [3], including the calculation of the Archimedes force. This model used the volume, length, and mass of the body segments of the participants on which the ballasts were placed, as well as the mass of the ballasts (placed at the CoM of the body segments) necessary to compensate for the Archimedes force until lunar gravity was reached. To increase the accuracy of calculating the volume of body segments of the participants, the photogrammetry method was used individually, with further calculation of the volume in Blender 2.8 software (Blender Foundation, Amsterdam, Netherlands). In Blender software, participant volumes were scaled according to measured participant height data. For the photogrammetry method, 1000 frames were taken with a high-resolution camera (12 MP) with a lens length (4.25 mm) and f/1.8, and the Agisoft Metashape 1.7.2 program (Agisoft LLC, St. Petersburg, Russia) with a DSM 114 = resolution 10 cm/pixel high-density cloud quality was used. To determine the accuracy of the 3D model, the volume of the participant was also measured using the water displacement method. The measurement difference was 5 cm ^3^.

To validate the lunar gravity model, the balance equation ΣF = 0 was used, with the Archimedes force for water conditions. For the balance equation, the reaction force of the support on the sitting part of the body was measured; it was balanced by the forces applied to the body above the pelvises of the participants. The details of the HG simulation model are described in the study by Volkova et al. [13] and the doctoral dissertation by Volkova [3].

The location of subjects’ CoM was determined using a vision-based markerless motion capture method under 1 g and 1/6 g for comparison reasons. Three video cameras with resolutions of 1920 × 1080 pixels at 30 Hz (4k condition), the sensitivity of an image sensor ISO 100 (Gopro 8, Woodman Labs, Inc. San Mateo, CA, USA) were used for the chosen method. All cameras were fixed around the participants at a distance of 2 m so that they could film all areas and sides of the body. For underwater experiments, the cameras were placed in waterproof boxes. To improve the recognition performance of participants, several conditions were met: namely, sufficient transparency of the water, good illumination, allowing the participants to see clearly, and specially selected clothing for the participants, mostly light tones with a dark pattern. All cameras were calibrated using software created by the Computer Vision Laboratory (CVLab) [33] at the Swiss Federal Institute of Technology in Lausanne. Openpose software (version 1.4.0) [34,35,36] was installed from GitHub to recognize participant’s 2D skeletons. To run this software, GPU (GEFORCE GTX 2080, Nvidia Corp, Santa Clara, CA, USA) was provided. Twenty-five critical key points of participants’ skeletons were recognized and saved in JSON format for each frame and camera. Using predictions from manually annotated data, and the data recognized by the software, joint recognition’s quality was examined. Markerless motion capture errors based on the percentage of correct key points (PCK) showed 100 for 1 g and 77 for 1/6 g across the four frames evaluated (see details in [31]). The results showed sufficient quality of recognition. Furthermore, a customized MATLAB script was utilized to determine the CoM. This script reads the JSON-formatted input data that receives after performing a triangulation.

Different factors, including the optical properties of water, the visibility of the participants and the scene, water resistance, the attachment of ballast to various parts of the body that affect the inertia of movement, and the ability to predict movement with the markerless motion capture method, including problems with camera synchronization, can affect the measured values.

### 2.2. Statistical Methods

The distribution of data was examined utilizing Microsoft Excel and the statistical software Stata 17 (SataCorp, College Station, TX, USA) and R software (R Core Team, Vienna, Austria). A statistical analysis using regression with correlation coefficient, standard errors, and *p*-value evaluation was carried out to determine the significance and nature of the relationship between gravity level and the mean CoM location along the proximodistal (Y) axis and along the anterior–posterior (Z) axis. Sex/gravity level was used as an independent variable, and CoM displacement along the proximodistal (Y)/anterior–posterior (Z) axis was used as the dependent variable.

Body segment masses and dimensions were assessed using statistical data obtained using the Plagenhof water immersion method [37]. Likewise, the CoM for the body segments was approximated using the statistical data of the same author. To illustrate, for the forearm, upper arm, and trunk, the CoM locations for males were positioned at 43%, 43.6%, and 63% of the segment and for females at 43.4%, 45.8%, and 56.9%, measured from the proximal end (see Figure 1b). From the hip joint to the shoulder, the length of the entire trunk was 100%.

The normality of the data was assessed through the application of the Shapiro–Wilk test (see Supplementary Materials Table S2). If the *p*-value exceeded 0.05, the data were normally distributed.

### 2.3. CoM Assessment

At the start, middle, and end of the experiment (the average of the first 5 s to 1 min), three measurements were collected for each participant. The duration of the experiment was determined by the endurance time (ET) of the participants. The ET is the period after the participant’s capacity to exert force has been lost when muscular tiredness begins to set in. Thus, the participants conducted the tasks until they felt muscle fatigue. Task duration data and CoM position data were taken into consideration as the analysis’s starting point in this study. Table 2 displays the data for the total ET for males and females for each task.

The hip joint keypoint (pelvis) was used as a reference point to determine the CoM. The measurements were taken in the sagittal plane. For each recorded frame, the CoM of the participants in the seated position was estimated automatically.

The following equation of the weighted sum of limb centers was used [38]:

(1) $$CoM=\frac{1}{M}\sum_{\left(i,j\right)\in Limbs}m_{ij}\frac{P_{i}+P_{j}}{k},$$

$\mathrm{with}\;m_{ij}$—segment mass connecting joints i,j;
$M=\underset{\left(i,j\right)\in Limbs}{\sum }m_{ij}$—total mass of all body segments;
$P_{i},\;P_{j}$—coordinates of keypoints of the body segment;
*k*—coefficient of the location of CoM of the segment, measured from the proximal end.

Coefficient *k* was determined using the Plagenhoff method [37], where, e.g., the forearm in male participants’ CoM was equal to 43% of the length of the segment.

## 3. Results

This analysis was conducted to assess the correlation between the CoM displacement of the participants and the gravity level. Table 3 shows the regression results for the mean values of the CoM displacement along the proximodistal (Y) axis and CoM displacement along the anterior–posterior (Z) axis around the pelvis. All analyses were conducted to contrast the impact of two different environments (1 g and 1/6 g) on the observed CoM.

The results are presented for males and females individually for the combined data sets for static and dynamic tasks.

**Figure 2:**
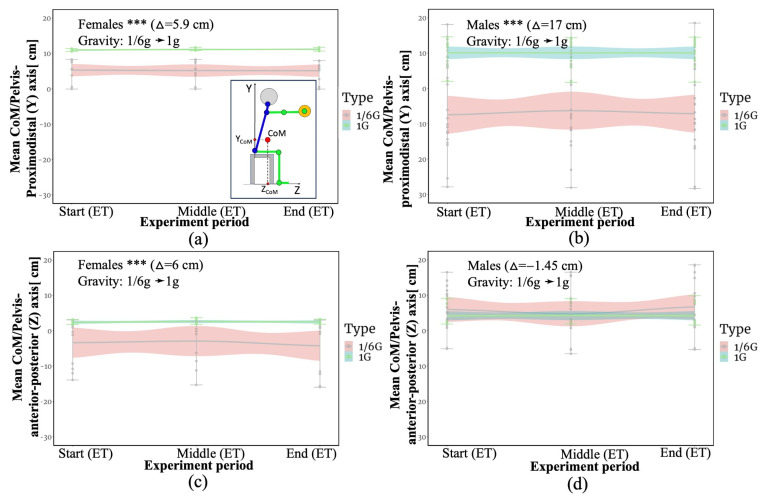
CoM mean values for static and dynamic tasks (cm): (**a**) CoM mean values for the proximodistal (Y) axis for females; (**b**) CoM mean values for the proximodistal (Y) axis for males; (**c**) CoM mean values on the anterior-posterior (Z) axis for males; (**d**) CoM mean values on the anterior–posterior (Z) axis for males. Gravity level is the transition from 1/6 g to 1 g of gravity. Δ—delta between CoM shift under 1/6 g and 1 g. *** *p* < 0.1. Measurements were taken at the beginning, middle, and end of the experiment, according to the endurance time (ET). CI, 95%—confidence interval.

Static and dynamic tasks were combined, since statistical calculations showed that if the type of task barely affects the results, then the sex of the experiment participants affects the change in the result. Tables S3 and S4 in the Supplementary Materials provide statistical findings on computed CoM for combined data sets for males and females. The results of the mean change in CoM coordinates along the proximodistal (Y) axis and the anterior–posterior (Z) axis relative to the pelvis for static and dynamic tasks together, considering participants’ ET, are presented in Figure 2 below.

Comparatively, the findings show that the CoM deviations vary greatly depending on the environment. The significance of this difference was demonstrated by the *p*-value, which reveals how significant the deviation is in general. The results of this study show that for static tasks and dynamic tasks for females and males, there was a significant correlation (*p* < 0.01) between the CoM displacement along the proximodistal (Y) axis and the level of gravity change (from 1/6 g to 1 g), with a correlation coefficient range of 5.9 cm to 17 cm (see Figure 2a,b). Only one significant correlation was found between the deviation of the CoM along the anterior–posterior (Z) axis and the change in the level of gravity (from 1/6 g to 1 g) for females performing static and dynamic tasks. The correlation coefficient is 6.02 cm (*p* < 0.01).

## 4. Discussion

This study emphasizes the critical importance of CoM for workplace ergonomics, optimizing the work environment, equipment, and tasks for worker safety and comfort, particularly in the HG environment. Understanding the CoM is vital for multiple reasons. Firstly, it determines stability, impacting accident and fall risks. Ergonomists analyze it to identify stability issues, enhance safety, and prevent accidents. Secondly, it influences posture and alignment, reducing musculoskeletal disease risk and ensuring comfort. Thirdly, it is crucial for safe lifting and movement. Ergonomic principles leverage CoM knowledge to guide lifting techniques, define load limits, and design equipment, thus reducing body strain. Considering the CoM enables the design of ergonomic strategies that minimize injury risks, enhance worker well-being, and boost work efficiency.

One significant aspect of this study is CoM prediction under simulated HG for examining human posture. The uniqueness of this study resides in the fact that for the first time, participant posture as indicated by CoM location was evaluated via the load under HG while seated. Additionally, simulating the biomechanics of the upper limbs at the workplace under HG is a recent study subject, and there is still a knowledge gap in this field, as previous studies focused on standing, walking, and jumping scenarios [10,11,12].

The results reveal that participants’ CoM tends to shift downward along the proximodistal (Y) axis and backward along the anterior–posterior (Z) axis in simulated lunar gravity (1/6th of Earth’s gravity), indicating a change in postural stability relative to 1 g conditions. This can probably be explained by the fact that when the body is under stress (under load, under the Earth’s gravity), participants tend to straighten their backs to a neutral sitting position. This theory is supported by the result obtained in Figure 2c, where the CoM of females tends to the vertical under the 1 g conditions. At the same time, the CoM shifts downwards, as the results of females and males show (see Figure 2a,b). The reverse situation is observed with simulated HG. This is likely because the bodies of participants become lighter in the simulated lunar gravity, and the postural stability and overall adaptability of the body to the environment changes. Even with a restraint system, participants’ bodies are less stable in simulated lunar gravity, so this should be considered when designing workplace design requirements. This is consistent with the spinal tilt in relation to the vertical results of the same participants previously reported in “Biomechanics at the workplace under hypogravity” Volkova [3] and Maillard [39]. According to the author, the findings on alterations in body positioning indicate a significant (*p* < 0.01) backward shift in torso inclination during seated positions while executing both static and dynamic tasks under HG. To the best of the author’s knowledge, there are no data on sitting and working at the workplace under HG, except for the indicated references. As a result of the difficulty in stabilizing the body, it is anticipated that the energy costs required in the Moon’s environment will be higher. This assumption agrees with [40]. However, this should still be validated. As the further direction of this study, the total work and metabolic energy while performing the tasks can be calculated for simulated 1/6 g and 1 g. To achieve this, the trajectory of body segments and CoM trajectory of the whole body are necessary. The trajectory of body segments as well as the CoM of the body can be potentially obtained with markerless motion capture.

The findings of the study also highlight the challenge of comprehending how the human body reacts to HG environments. Despite the study’s insightful findings, there is a need for more research, particularly regarding the complex relationships between perception and body tilt in simulated HG. Particularly, changes in participants’ tactile, vestibular, or visual perception and body tilt under simulated HG can be assessed. This area is still under investigation and there are many ambiguities [41]. According to Harris [41], to understand this, it is necessary to conduct multisensory observation combining visual, vestibular, somatosensory, and proprioceptive studies. Another author [42] found a problem in the perception of HG related to the “G-shortage” illusion that could lead to the underestimation of roll tilt.

One of the notable results of this study is the application of an indirect method of data collection, namely markerless motion capture techniques in aquatic environments. It was found that the use of markerless motion capture techniques could increase experiment efficiency and improve data collection. This is particularly beneficial for the non-invasive studies of participants, such as extensive water trials. Cost-effectiveness and non-invasiveness advantages were also reported by Shipley and Brumberg [43] and Lam et al. [44]. Moreover, the markerless system may be a suitable alternative to marker-based solutions due to the comparable accuracy of results. This statement is in agreement with Kanko et al. [45]. These solutions are substantially less expensive than sensor-based approaches, which can greatly enhance the amount of data collected and, consequently, the findings. Also, these techniques can be used on projects that are still in the design phase. For further experiments, the use of a single camera with LiDAR in underwater conditions might be used.

There are several limitations of this study. Firstly, the research focused on a scant number of motions. While repetitive tasks and other categories of static tasks can also be analyzed, this study concentrated on the investigation of only static and dynamic movements. This is due to the volume of video data generated and the difficulty of processing the information gathered from the three cameras used in the postural study.

Another limitation relates to the chosen markerless motion capture technique. The results may be affected by errors encountered during underwater video recording related to the optical properties of water and the visibility of participants and the scene, including problems with camera synchronization. From an injury biomechanics perspective, the combined assessment of posture and body shape can be used to improve the level of accuracy [46].

The results of the study should also be interpreted in the context of the experimental setup and the software used: a minor error in the calculation of the CoM may arise due to the calculation of the volume of the torso, the minimal influence of the moments of inertia of the distributed operational weights on parts of the participant’s body, as well as errors arising from water resistance when the participant performs movements.

While the study provides valuable insights into simulated HG conditions, to ensure the validity of the results, they should be verified in real-world settings, for instance, a parabolic flight campaign tailored to specific conditions.

## 5. Conclusions

In conclusion, this study emphasizes the significance of CoM in workplace ergonomics and its relevance to ensuring worker safety, comfort, and productivity. It expands the knowledge of CoM dynamics under simulated HG while seated, illuminating potential difficulties and considerations for future lunar and Martian base designs. For these types of studies, the use of markerless motion capture techniques provides a cost-effective and efficient method of data collection.

## Figures and Tables

**Table 1:** Descriptive statistics of the participants. Modified and adapted from [3].

Study Parameters	Total (N = 32)		Male (N = 18)	Female (N = 14)	*p*-Value
	Mean (SD) ^1^	Min/Max	Mean (SD)	Mean (SD)	
Age (year)	33.59 (8.16)	25/55	34 (9.62)	33.07 (6.11)	0.742
Height (m)	1.75 (0.11)	1.54/1.95	1.83 (0.07)	1.66 (0.06)	˂0.001
Body mass (kg)	71.22 (17.01)	43.8/114.10	82.92 (13.02)	56.19 (5.95)	˂0.001
Upper arm (m) ^2^	0.34 (0.04)	0.25/0.40	0.35 (0.33)	0.32 (0.03)	0.007
Forearm (m) ^2^	0.28 (0.03)	0.20/0.33	0.30 (0.02)	0.25 (0.02)	˂0.001
Torso volume (dm^3^)	37.00 (11.00)	28.00/61.00	44.71 (6.93)	27.00 (4.42)	˂0.001
Upper arm volume (dm^3^)	2.00 (0.80)	0.8/3.8	2.70 (0.67)	1.48 (0.38)	˂0.001
Forearm volume (dm^3^)	1.00 (0.30)	0.4/2.00	1.37 (0.20)	0.72 (0.16)	˂0.001

^1^ SD—standard deviation. ^2^ Upper arm (m)*—*the distance between the keypoints of the shoulder and the upper part of the forearm. Forearm (m)*—*the distance between keypoints of the lower part of the upper arm and the upper part of the hand.

**Table 2:** Endurance time (ET) (min) of the participants. Tasks: static and dynamic are presented separately. Males/females. Modified and adapted from [13].

Gravity	mn ^1^ ET (min) for 1 kg Load	mn ET (min) for 3 kg Load
	Static (22 participants)	
1 g	1.67/0.95	0.85/0.34
1/6 g	7.73/6.21	2.65/0.65
	Dynamic (25 participants)	
1 g	1.30/0.80	0.79/0.35
1/6 g	14.93/9.34	2.16/0.82

^1^ mn—mean value.

**Table 3:** The mean of CoM coordinates changes along the proximodistal (Y) axis and the anterior–posterior (Z) axis relative to the pelvis for static and dynamic tasks together [deg.]. Males (M); females (F). Gravity level (G-level) refers to the gravity change from 1/6 g to 1 g. Adapted from [3].

Variable	CoM—Proximodistal Axis—Pelvis		CoM—Anterior–Posterior Axis—Pelvis	
	M	F	M	F
G-level	17.06 *** ^1^ (1.58) ^2^	5.87 *** (0.57)	−1.45 (1.50)	6.02 *** (1.43)
Task	3.17 ** (1.59)	−0.94(0.57)	−2.17 (1.50)	0.44 (1.41)
Constant ^3^	−8.47 *** (1.35)	5.70 *** (0.48)	6.67 *** (1.28)	−3.77 *** (1.20)
Observations	114	56	114	56
R ^2^	0.52	0.67	0.03	0.25
Adjusted R ^2^	0.51	0.66	0.01	0.22
Residual Std. Error	8.45	2.11	8.01	5.26
F Statistic	60.01 ***	54.28 ***	1.51	8.91 ***

^1^ *** *p* < 0.1; ** *p* < 0.05. ^2^ The standard error is also shown by the indicator between the brackets. ^3^ Constant—the correlation that cannot be explained by the explanatory variables: g-level and task. This is common for all the observations.

## Data Availability

The data presented in this study are available in https://figshare.com/s/3ad227d2053821069fc9, accessed on 22 September 2023.

## Supplementary Materials

The following supporting information can be downloaded at: https://www.mdpi.com/article/10.3390/bioengineering10101221/s1, Figure S1: The 2020 Physical Activity Questionnaire (PAR-Q+) [32]; Table S1: G*Power sample size analysis results. F tests—ANOVA: repeated measures, with-in-between interaction. A significance level of α = 0.05 was established. The specified benchmarks, as outlined in [47], were 0.2 (indicating a small effect), 0.5 (representing a moderate effect), and 0.8 (indicating a large effect) between the experimental groups; Table S2. Shapiro–Wilk normality test results for females/males for center of mass (CoM) study experiment. W-test statistic; Table S3. The mean CoM coordinates along the anterior-posterior (Z) axis/Pelvis for males and females together (cm). Gravity level (G-level) means gravity change from 1⁄6 g to 1 g. *** *p* < 0.1; ** *p* < 0.05. The standard error in the calculations is also shown by the indicator between the brackets. Constant—the correlation that cannot be explained by the explanatory variables: g-level and task. This is common for all the observations. Adapted from [3]; Table S4: The mean CoM coordinates along the proximodistal (Y) axis/pelvis for males and females together (cm). Gravity level (G-level) means gravity change from 1/6 g to 1 g. *** *p* < 0.1. The standard error in the calculations is also shown by the indicator between the brackets. Constant—the correlation that cannot be explained by the explanatory variables: g-level and task. This is common for all the observations. Adapted from [3].
