# Supplementary material for: The Three-Dimensional Body Center of Mass at the Workplace under Hypogravity
Source: Bioengineering (Basel). 2023 Oct 19;10(10):1221. doi: 10.3390/bioengineering10101221 (PMC10604834; doi:10.3390/bioengineering10101221)
Supplement: Supplementary file 1 [file bioengineering-10-01221-s001.zip › bioengineering-2652534-supplementary.pdf]

Supplementary materials

The Three-Dimensional Body Center of Mass at the Workplace under Hypogravity

Tatiana Maillard

Space Innovation, Swiss Federal Institute of Technology Lausanne; tatiana.maillard@alumni.epfl.ch

Supplementary Materials:

Figure S1: The 2020 Physical Activity Questionnaire (PAR-Q+) [32]

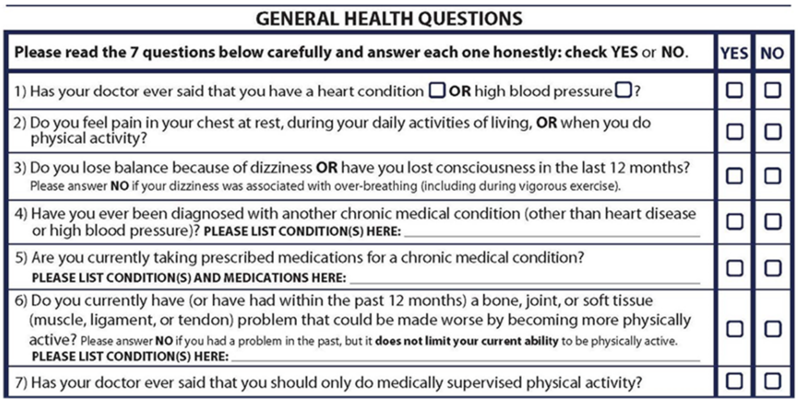

GENERAL HEALTH QUESTIONS

**Please read the 7 questions below carefully and answer each one honestly: check YES or NO.**	YES	NO
1) Has your doctor ever said that you have a heart condition ☐ **OR** high blood pressure ☐?	☐	☐
2) Do you feel pain in your chest at rest, during your daily activities of living, **OR** when you do physical activity?	☐	☐
3) Do you lose balance because of dizziness **OR** have you lost consciousness in the last 12 months? Please answer **NO** if your dizziness was associated with over-breathing (including during vigorous exercise).	☐	☐
4) Have you ever been diagnosed with another chronic medical condition (other than heart disease or high blood pressure)? **PLEASE LIST CONDITION(S) HERE:** ______	☐	☐
5) Are you currently taking prescribed medications for a chronic medical condition? **PLEASE LIST CONDITION(S) AND MEDICATIONS HERE:** ______	☐	☐
6) Do you currently have (or have had within the past 12 months) a bone, joint, or soft tissue (muscle, ligament, or tendon) problem that could be made worse by becoming more physically active? Please answer **NO** if you had a problem in the past, but it **does not limit your current ability** to be physically active. **PLEASE LIST CONDITION(S) HERE:** ______	☐	☐
7) Has your doctor ever said that you should only do medically supervised physical activity?	☐	☐

Table S1: G*Power sample size analysis results. F tests – ANOVA: repeated measures, within-between interaction. A significance level of α = .05 was established.

Input	
Effect size f	0.25
α error prob	0.05
Power (1-β error prob)	0.8
Number of groups	2
Number of measurements	3
Correlation among repeated measures	0.8
Nonsphericity correction e	1
**Output**	
Noncentrality parameter λ	11.2500000
Critical F	3.4928285
Numerator df	2.0000000
Denominator df	20.0000000
Total sample size[1]	12
Actual power	0.8005308

[1] The specified benchmarks, as outlined in [47], were 0.2 (indicating a small effect), 0.5 (representing a moderate effect), and 0.8 (indicating a large effect) between the experimental groups. When examining the impact of environmental factors on postural variables during tasks, a minimum of twelve participants is necessary to achieve an actual power of 0.80.

Table S2. Shapiro-Wilk normality test results for females/males for center of mass (CoM) study experiment. W-test statistic

Task	W	p-value
CoM -Y axis - Pelvic -1 g	0.96/0.88	0.46/0.01
CoM -Y axis - Pelvic - 1/6 g	0.85/0.95	0.01/0.03
CoM – Z axis - Pelvic -1 g	0.92/0.72	0.09/0.01
CoM -Z axis - Pelvic - 1/6 g	0.80/0.95	0.01/0.07

Table S3. The mean CoM coordinates along the anterior-posterior (Z) axis/pelvis for males and females together (cm). Gravity level (G-level) means gravity change from 1⁄6 g to 1 g. ***p<0.1; **p<0.05. The standard error in the calculations is also shown by the indicator between the brackets. Constant – the correlation that cannot be explained by the explanatory variables: g-level and task. This is common for all the observations. Adapted from [3].

Variable	CoM - anterior-posterior (Z) axis/pelvis	
	Static	Dynamic
G-level	2.71 (2.00)	-0.74 (1.14)
Gender	-4.43** (2.08)	-7.23***(1.24)
Constant[1]	0.65 (1.38)	3.91***(0.93)
Observations	84	86
R^2	0.08	0.29
Adjusted R^2	0.06	0.27
Residual Std. Error	9.08	5.26
F Statistic	3.50**	17.04***

Table S4. The mean CoM coordinates along the proximodistal (Y) axis/pelvis for males and females together (cm). Gravity level (G-level) means gravity change from 1⁄6 g to 1 g. ***p<0.1. The standard error in the calculations is also shown by the indicator between the brackets. Constant – the correlation that cannot be explained by the explanatory variables: g-level and task. This is common for all the observations. Adapted from [3].

Variable	CoM - proximodistal (Y)/ pelvis	
	Static	Dynamic
G-level	11.13*** (1.74)	15.71*** (1.51)
Gender	4.99*** (1.81)	9.33***(1.64)
Constant[1]	-2.34 (1.38)	-7.80***(1.17)
Observations	84	86
R^2	0.36	0.62
Adjusted R^2	0.34	0.61
Residual Std. Error	7.90	6.97
F Statistic	22.83***	66.48***
